# Bone Health and Its Positive Relationships with Body Composition in Malaysian Schoolchildren: Findings from a Cross-Sectional Study

**DOI:** 10.3390/children8070569

**Published:** 2021-07-02

**Authors:** Hui Chin Koo, Geok Pei Lim, Satvinder Kaur, Kai Quin Chan, Keh En Chan, Casey Chung, Michelle Wong, Ugunesh Danaselvam

**Affiliations:** 1 Department of Bioscience, Faculty of Applied Sciences, Tunku Abdul Rahman University College, Kuala Lumpur 53300, Malaysia; geokpeilim@hotmail.com (G.P.L.); chankq@tarc.edu.my (K.Q.C.); chanke-wa11@student.tarc.edu.my (K.E.C.); chhungc-wl17@student.tarc.edu.my (C.C.); michellewst-wp16@student.tarc.edu.my (M.W.); uguneshd-wp16@student.tarc.edu.my (U.D.); 2 Faculty of Applied Sciences, UCSI University, Kuala Lumpur 56000, Malaysia; satvinderkaur@ucsiuniversity.edu.my

**Keywords:** body composition, bone health, Malaysia, schoolchildren

## Abstract

Background: Optimal bone health is vital in children to prevent osteoporosis later in life, and body composition plays a crucial role in it. However, the literature reports contradictory results when considering the relationship between body composition and bone health in children. This study aimed to examine the bone health and its relationship with body composition in Malaysian schoolchildren. Methods: In this cross sectional study, body composition data (weight, height, body fat percentage [% fat], fat mass, fat free mass, visceral fat, waist circumference [WC] and body mass index-for-age [BMI z-score]) and bone health data (Z-score and broadband ultrasound attenuation [BUA]) were collected from 415 schoolchildren aged 9–12 years, cluster sampled from randomly selected primary schools in Kuala Lumpur, Malaysia. Results: Girls generally had significantly higher height, body fat percentage, fat mass, visceral fat and Z-score as compared to boys. A steady increase of the mean BUA value was observed with increasing age in both sexes. The mean BUA value of the present study across the population was significantly higher than that of schoolchildren from Nigeria (*p* < 0.001), Colombia (*p* < 0.001) and Spain (*p* = 0.002). Significant positive correlations were found between all the body composition variables and bone outcome variables across the population. Further, BUA was significantly correlated with weight (β = 0.172; *p* = 0.001), height (β = 0.299; *p* < 0.001), % fat (β = 0.131; *p* = 0.007), fat mass (β = 0.130; *p* = 0.007), fat free mass (β = 0.209; *p* < 0.001), visceral fat (β = 0.127, *p* = 0.008), WC (β = 0.165; *p* = 0.001) and BMI z-score (β = 0.162; *p* = 0.001), after controlling for sex, age and ethnicity. Similarly, after confounders adjusted, Z-score was significantly predicted by weight (β = 0.160; *p* = 0.001), height (β = 0.310; *p* < 0.001), % fat (β = 0.104; *p* = 0.032), fat mass (β = 0.107; *p* = 0.026), fat free mass (β = 0.218; *p* < 0.001), visceral fat (β = 0.107, *p* = 0.026), WC (β = 0.145; *p* = 0.002) and BMI z-score (β = 0.150; *p* = 0.002). Conclusions: Our findings have revealed that body composition variables were positive correlated with bone outcome variables, suggesting that adipose tissue acts to stimulate bone growth. Further clinical and molecular studies in the future is recommended to fully illustrate the complex interactions between adiposity and bone health.

## 1. Introduction

Osteoporosis prevention, which is usually associated with a geriatric disorder, may now be considered the legitimate domain in children population [1]. With the advent of improved diagnostic and treatment options, threats to bone health in children are receiving increased attention. The underlying assumption, widely shared despite the absence of evidence, is that peak bone mass gradually increases during childhood and adolescence, accounting for about half of the bone mass achieved in adulthood [2]. Cellular activity within the bone microenvironment favors net bone gain with the best increases in bone mass happening during puberty, a period of peak bone mass accrual; which is very important determinant of osteoporosis later in life [3]. Whilst up to 80% of peak bone mass is genetically determined, the remaining 20% is modulated by environmental factors [1]. Therefore, childhood may be a pivotal period to identify factors that affect bone health.

Childhood obesity has gained more and more attention from healthcare professionals in developing and developed countries [4]. The global prevalence of childhood obesity rose by 47.1% from 1980 to 2013 [5]. If current trends prevail globally, the number of overweight and obese children will increase to 70 million by 2025 [6]. Malaysian bears the burden of rising prevalence in childhood obesity too. In Malaysia, the prevalence of overweight and obesity among children aged 5–17 years has increased dramatically, from 11.9% in 2015 [7] to over 14.8% in 2019 [8]. Although childhood obesity is not defined as a disease, obese children are at risk of severe obesity in adulthood, metabolic syndrome and health problems later in life [9].

A number of studies aimed to analyze the relationship between body composition and bone health were performed in adult population, which have demonstrated a positive relationship between body mass index (BMI) and bone mineral density (BMD) [10]. The effect of body composition on bone health in children population are less well studied; but since childhood may be a critical life-stage for skeletal mineralization, it is important to grasp how body composition in childhood may affect bone health. Studies to evaluate the effect of body composition on bone health in children have reported inconsistent results. Growing evidence demonstrates that overweight and obese children are over-represented in fracture groups [11]. It has raised concerns that the worldwide rise in childhood obesity could have a major impact on bone health within the short- and long-term. On the other hand, it has been reported an increased rate of bone mineral density (BMD) in children and adolescents with obesity, indicating that the adipose tissue exerts a positive effect on bone structure [12]. Kindler [13] has shown a similar outcome, where overweight and obese children have greater bone mass and strength compared to normal weight children.

In Malaysia, published journal articles on bone health are still scarce among the children population. Researches on the bone health have primarily focused on Malaysian postmenopausal women [14] and adult populations [15]. Moreover, no published research has investigated the relationship between bone health and body composition in Malaysian children. The objectives of the present study were: (1) to determine the bone health among Malaysian schoolchildren; and (2) to examine the linkages between the bone health with z-scores for body mass index-for-age (BMI z-score), body fat percentage, fat mass, fat free mass, visceral fat and waist circumference; but not to claim the causality. We hypothesized that bone outcome variables would have positive relationships with body composition in Malaysian schoolchildren.

## 2. Methods

### 2.1. Participants and Study Design

A sample of 415 healthy Malaysian schoolchildren aged 9–12 years participated in this cross sectional study. All the schoolchildren who fulfilled the inclusion criteria from the three selected national primary schools were invited to participate in the study, by using cluster sampling method.

Considering that Kuala Lumpur has 41,872 schoolchildren aged 9–12 years, the estimated sample size for this study was 381 schoolchildren. This calculation was performed by setting the predicted prevalence of 50% for a confidence interval of 95%, and a relative precision of 5% [16]. To cater for loss of information in case of incomplete data, 10% was added to the sample. Inclusion criteria were: (1) healthy Malaysian schoolchildren aged 9–12 years, regardless of ethnicity; (2) ability to read, write and understand Bahasa Malaysia; and (3) present in the school on the day of data collection, with obtained written parental consent. Exclusion criteria were: (1) non-attendance on the data collection day; (2) use of drugs or medication that interferes with bone density or body composition; and (3) presence of a physical disability that could affect the assessment.

All procedures performed in the study were in accordance with the ethical standards of the Tunku Abdul Rahman University College Ethics Committee, which approved the study protocol (TAR UC/EC/2018/01-3). Permission to carry out data collection was granted by the Ministry of Education Malaysia and the Kuala Lumpur Federal Territory Education Department. A comprehensive written description of the nature and purpose of the study, and its experimental risks was given to the schoolchildren and their parents/guardians. Written informed consent was obtained from the parents prior to the data collection. Verbal assent was obtained before schoolchildren participated in the study too, to enable acquisition of bone health, anthropometric and adiposity measurements.

### 2.2. Bone Health Measurement

Bone health was evaluated using PENGASUS calcaneal quantitative ultrasound (QUS) device (Medilink, Mauguio, France), which measured bone properties at the calcaneus (bone of the heel). The technique is based on transmission of ultrasound signals, using a pair of non-focused transducers, with the mean frequency of 1.6 MHz. The coupling between the probes and the foot was performed using standard ultrasound gel. Ultrasound amplitude, broadband ultrasound attenuation (BUA, dB/MHz) and speed of sound (SOS, m/s) were calculated from the ultrasound signal transmitted through the heel. Measurements of QUS were obtained by study researchers using standard operating procedures as specified in the manufacturer’s operators manual [17]. Daily calibration was done using a phantom block prior to every scan, to ensure proper coupling, water level and unit temperature. All measurements were performed using foot shim provided by the manufacturer for positioning assistance. The size, location of the region of interest and the proper positioning of the foot, were determined using the QUS device’s moveable region of interest feature and the real time QUS image [18]. Measurement outcomes including BUA, SOS, T-score, Z-score, heel thickness and osteoporosis status. All the schoolchildren were measured twice, to maximize the accuracy of outcomes.

According to the manufacturer’s operators manual [17], BUA is the main parameter measured by PEGASUS, the SOS value is computed but it is not used for any analyses. The recommended reference groups for the T-scores are young gender matched populations (20 to 40 years old) at peak bone mass; whereas, the Z-scores are derived from age-matched reference populations, expressed in number of standard deviations from the reference groups [17]. The information provided in the manufacturer’s operators manual [17] are in line with several reviews, where BUA is the most common variable reflecting ultrasound attenuation through bone by calcaneal QUS devices [19], the SOS measurements at the radius is not appropriate for assessing bone quality status among the children aged 6 to 12 years [20], and Z-score is recommended for diagnosis in children population [21]. Therefore, results of BUA and Z-score were applied in our data analyses for bone health measurements.

To date, no universal cut-off point exists for bone health categories using the QUS technique in schoolchildren. Despite the fact the International Society of Clinical Densitometry (ISCD) has recommended the use of a Z-score cut off point of −2 to define low bone density in children, this does not apply equally to techniques other than dual energy X-ray absorptiometry (DXA) [22]. Therefore, the present study does not categorize the schoolchildren according to bone health status.

### 2.3. Body Composition

The body composition of each child was measured twice, with the schoolchildren wearing a light school uniform. Any items that could affect the readings were removed from their pockets prior to the measurements. Right after the electrode of the scale was cleaned, the schoolchildren stood bare-foot on the footpad shapes of the electrode guides. Weight, body fat percentage, fat mass, fat free mass and visceral fat were measured using a calibrated InBody body composition analyzer Model 270 (InBody Co., Seoul, Korea). It is a bioimpedance device, comprise of two electrodes. Impedance is calculated by using two formulas: (1) calculating the volume of a cylinder (volume = length × area); and (2) characteristic of impedance, where impedance is inversely proportional to cross-sectional area and directly proportional to length. By knowing the impedance and the length of the cylinder, the volume of total body water will be measured.

The weight, body fat percentage, fat mass, fat free mass and visceral fat were recorded to the nearest 0.1 kg, 0.1%, 0.1 kg, 0.1 kg and 1 unit, respectively. Waist circumference of the schoolchildren was measured twice, based on the WHO [23] standard protocol, which measured the waist circumference at the mid-point between the highest point of the iliac crease and the last floating rib. Waist circumference was measured using a Lufkin tape model W606PM (Apex Tool Group, Sparks, MD, USA), to the nearest 0.1 cm. Height was measured twice, using a calibrated InBody digital free-standing stadiometer Model BSM170 (InBody Co., Seoul, Korea). The height was recorded to the nearest 0.1 cm. BMI z-score was determined based on the World Health Organization (WHO) AnthroPlus version 1.0.3 (World Health Organization, Geneva, Switzerland) software. The reported values of the Body composition were the average values from the two readings taken.

### 2.4. Statistical Analyses

Statistical Package for the Social Sciences (SPSS) version 22.0 (IBM SPSS Statistics 2014, IBM Corp., Armonk, NY, USA) was used for statistical analyses. The Kolmogorov-Smirnov test was run to examine the normality for each variable. Mean ± standard deviation and median ± interquartile range being reported for all the variables. Sex-group differences in the weight, body fat percentage, visceral fat, fat mass, fat free mass, BMI z-score and waist circumference data were analyzed by Mann Whitney Test. We also analyzed sex-group differences in the height, BUA and Z-score data by using Independent t-test. One-way Anova and Tukey tests were applied to compare the mean BUA value for schoolchildren from the present study (Malaysia), Singapore [24], Colombia [25], Nigeria [26] and Spain [27]. Unadjusted correlations of body composition variables with bone outcome variables were tested by using Pearson Correlation coefficient test. A significance level of *p* < 0.05 was applied to fix a cut-off for further analysis with each variable. Multiple linear regression analysis was then used to determine the extent of the relationships between bone outcome variables and each of the body composition variables with a significant correlation, while adjusting several confounders, including sex, ethnicity and age. Ethnicity was coded as a dummy variable, where “0” represents Malay and “1” represents Chinese, Indian and other ethnicity. According to Vatcheva [28], multicollinearity occurs when at least two significantly correlated predictors/independent variables are assessed concurrently in a regression model. Our body composition variables are significantly correlated with each another; hence, each of the body composition variables were run individually with its own model, with twelve models being created in total. BUA and Z-score were dependent variables. Independent variables consisted of weight, height, body fat percentage, visceral fat, fat mass, fat free mass, waist circumference and BMI z-score. All the model assumptions, e.g., multicollinearity, linear relationship, independent errors, multivariate normality, independent errors and homoscedasticity were conducted and fulfilled, prior to the multiple linear regression performance. *p* < 0.05 (two-sided) was considered statistically significant.

## 3. Results

Demographic characteristics, body composition and bone outcome variables of schoolchildren are listed in Table 1. A total of 415 healthy Malaysian schoolchildren (196 boys and 219 girls) were recruited from Kuala Lumpur, Malaysia. The median age of the schoolchildren was 10.8 years and the majority of them were Malay ethnicity (66.7%; *n* = 277). The ethnicity distribution was comparable to the percentage derived from the Malaysia Population and Housing Census 2019. Comparing between boys and girls, no significant differences were shown in the potential confounders (ethnicity and age); however, there were significant differences in body composition variables between the sexes. Girls generally had significantly higher height (143.5 cm vs. 141.8 cm; *p* < 0.05), body fat percentage (25.2% vs. 22.5%; *p* < 0.001), fat mass (10.5 kg vs. 9.7 kg; *p* = 0.001) and visceral fat (5 vs. 4; *p* < 0.001) as compared to boys. The average of weight, waist circumference and BMI z-score were similar in the both sexes. The bone outcome variables revealed no significant differences in the BUA outcome between both sexes; but significant differences were shown in the Z-score, which was higher in girls (1.7 vs. 1.1; *p* < 0.001).

We have compared the BUA outcome from the present study with similar age-group schoolchildren from Singapore [24], Colombia [25], Nigeria [26] and Spain [27]. As shown in Table 2, a steady increase of the mean BUA value was observed with increasing age in both sexes, for all the five countries. The mean BUA value of the present study across the population was significantly higher than that of schoolchildren from Nigeria (*p* < 0.001), Colombia (*p* < 0.001) and Spain (*p* = 0.002). Singaporean schoolchildren had significantly higher BUA as compared to Nigerian (*p* = 0.008) and Colombian (*p* < 0.001) schoolchildren too.

With regard to the unadjusted correlations between bone health and body composition variables, Table 3 showed that all body composition variables were positively correlated with bone outcome variables across the population. The findings from the simple linear analyses were further explored using multiple linear regression, with ethnicity, age and sex adjusted. The outcomes are summarized in Table 4. A total of sixteen individual multiple linear regression models were conducted to predict BUA and Z-score, using measures of body composition. 

All eight body composition variables were significantly correlated with BUA and Z-score, after controlling for ethnicity, age and sex. We found that BUA increased by 0.172, 0.299, 0.131, 0.130, 0.209, 0.127, 0.165, 0.162 SD, respectively; for every one SD increased in weight, height, body fat percentage, fat mass, fat free mass, visceral fat, waist circumference and BMI z-score, respectively. Similar findings were observed in Z-score analyses. With increasing of every one SD in weight, height, body fat percentage, visceral fat, waist circumference and BMI z-score, respectively; Z-score would increase 0.160, 0.310, 0.104, 0.107, 0.218, 0.107, 0.145 and 0.150 SD, respectively.

## 4. Discussion

The present study aimed to determine the bone health and its relationships with body composition among Malaysian schoolchildren aged 9–12 years. Our study also provided sex-specific values of the body composition and bone health parameters in Malaysian schoolchildren. It delivers novel data and discloses that Malaysian schoolchildren had significantly higher BUA than Colombian [25], Nigerian [26] and Spanish [27] schoolchildren. We further found that all the body composition variables performed optimally in predicting BUA and Z-score in schoolchildren. Given that obesity [6] is one of the major public health issues in child populations, and a previous study suggested that osteoporosis occurs in adulthood may be originating in childhood years [29], we believe the findings of the present study may provide an insight for future research in an effort to further clinical and molecular studies, to investigate the critical threshold of excess fat mass needs to be reached to reverse the potentially positive effects of fat on bone, and fully illustrate the complex interactions between adiposity and bone heath.

Our findings provide significant support for the view that girls have higher body fat percentage, fat mass and visceral fat, as regardless of their overall body weight, BMI z-score and waist circumference were comparable. It is in line with a review, where girls have shown an increase in body fat throughout the pubertal transition; sex-specific difference in fat distribution has been observed prior to puberty, and becomes more prominent in early of puberty [30]. In a cross sectional study conducted among 899 Vietnamese adolescents aged 11–14 years, body fat percentage was significantly higher in girls compared to boys. In addition, a descending trend in body fat percentage was shown in boys with increasing age, compared to girls where their body fat percentage was slightly increased with age [31]. This scenario can also be well-described by biological evidence and metabolic responses; where the differences has been attributed to sexual dimorphism in the sex hormone receptors expression, circulating of leptin levels and the differential expression of several proteins in the district adipose depots [32].

Subsequent findings of the present study have shown that height and Z-score were significantly higher in girls. Bone health assessment in children is more challenging than in adults, and requires further consideration of the puberty and growth impacts on bone density [33]. It is interpreted by using a Z-score, the number of standard deviation from the mean of age-matched reference [34]. On average, girls begin their pubertal growth spurt at age 9–10 years and boys at age 11–12 years [35]. A taller child should have larger bones and therefore, better bone density than those who are shorter [34]. It is plausible that differences in height and Z-score in the present study, in part, explain the earlier onset of peak height velocity in girls corresponding to an earlier onset of puberty development [35]. Our findings are supported by a published journal, where the bone density of both sexes was similarly increased until 9 years, followed by a higher increment of bone density presented in girls as compared to boys; authors of the published journal article suggested that the estrogen stimulation effect on the trabecular bone is responsible for this difference [36].

Surprisingly, Malaysian schoolchildren demonstrated the highest mean value of BUA for both sexes, compared to schoolchildren from Singapore [24], Colombia [25], Nigeria [26] and Spain [27]. Further, the mean BUA value of the present study across the population was significantly higher than that of schoolchildren from Nigeria, Colombia and Spain. There are various reasons for these differences. One such reason, bone health has been attributed to the lifestyle factors, e.g., physical activity level and sunlight exposure [34]. Malaysia, Singapore, Colombia and Nigeria are tropical or sub-tropical countries located near to the equator, with almost all year round of sunshine for cutaneous synthesis of vitamin D. Vitamin D plays a crucial role in bone health maintenance and metabolism [37]. Although prevalent vitamin D insufficiency has been reported in the Malaysian children population aged 0.5–12.9 years [38], however, the prevalence (47.5% vs. 60.4%) is much lower as compared to children aged 3.1–15.4 years from Spain, a non-tropical country [39]. Secondly, socio-economic and dietary factors also contributes to the observed differences in bone health [40]. Nigerian children are at particular risk of malnutrition and osteoporosis, considering Nigeria is one of the poorer countries, and the scenario worsen by the recent disasters that have increased internally displaced persons [26]). Thirdly, bone health differences in several regions might be accounted for by dissimilarities in various types of anthropometric measurements; these variables are important determinants of bone health, and may show a discrepancy between ethnicities and populations [25]. A further consideration in the present study comparing the bone health between Malaysian and Colombian schoolchildren [25], one extra analyses to present the height measurement according to sex- and age-matched groups was conducted (Table A1 in Appendix A). Schoolchildren from the present study showed higher height measurement for both sexes, and as a result will present with a greater bone density [34]. Other factors e.g., medications, medical conditions, genetic and ethnicity may also contribute to the differences in bone health [41].

The debate as to the optimal body composition for predicting bone health in schoolchildren had been the topic of researches. Notwithstanding a substantial body of bone mineral density data, the relationship between adiposity and bone outcomes remains conflicting. Our findings demonstrated that body composition variables, including weight, height, body fat percentage, visceral fat and waist circumference; were positively correlated with bone outcome variables, including BUA and Z-score. Further, these body composition variables were predictors of bone outcome variables. Our findings agree with those of a cohort study conducted among 7333 healthy Caucasian children aged 9 years [42]. These results were in conformity with findings a systematic review [43]. Studies support the evidence that in early childhood years, adiposity presents a structural advantage to the bone health [44]. On the contrary, a study conducted among 377 Brazilian adolescents aged 10–19 years demonstrated an inversely linear relationship between bone density and body mass index [45]. It is worth mentioning that studies among obese pre-pubertal children aged 9–12 years from France [46] and overweight children aged 3–19 years from New Zealand [47] also demonstrated negative relationships. However, it brings to the issue that the studies showed positive relationships outcomes, consisted of healthy children; instead of those with overweight and obese. Inconsistent outcomes between studies might be due to the differences in duration of obesity, severity and age [48].

Taken together, the contradictory outcomes can be explained by several potential mechanisms. Mechanical loading is the direct pathway for weight to affect bone health. Adiposity may greater the mechanical load, it may help in stimulating bone formation [49]. Besides mechanical loading, adiposity may demonstrate a positive relationship with bone health, by producing estrogens from steroid precursors, as well as increasing the insulin and leptin levels in the circulation; these process may help in stimulating bone formation [49]. Nonetheless, adiposity may contribute negative effects on the bone health too, by producing interleukin 6 and tumor necrosis factor [49]. Furthermore, “fat threshold” might be another attribution of conflicting outcomes. Explicitly, the negative relationship between childhood obesity and bone health only happened if adiposity reached excessive levels in a child, or following the evolution of an adverse metabolic profile [49]. Several studies propose discrepancy effects of adiposity on bone relative to age too. A review showed a positive relationship between adiposity and bone health in young children, which begins to attenuate later in childhood, and then showed the contrary relationship in adolescence and could potentially persist into adulthood [3]. Ethnicity and lifestyle factors may contribute to the changing relationship between adiposity and bone health too [41]. Hence, review suggested that inconsistent outcomes demonstrating the effect of adiposity on bone health may be partly explained by the stage of pubertal development and heterogeneous age ranges within and between studies [30].

The present study has a number of strengths. To the best of our knowledge, it is the first study to assess the relationship between bone health and body composition in Malaysian schoolchildren. In addition, we were able to adjust for several potential confounders, including sex, age and ethnicity. All the body composition variables were run individually with its own model, as body composition variables are significantly correlated with each another. This method may prevent the multicollinearity occurs when at least two significantly correlated predictors/independent variables are assessed simultaneously in a regression model [28]. Further, bone health was evaluated using QUS device. Advantages of this method over others are that it can be readily performed in the school setting, more accessible due to its portability, ease of operation, low cost, zero-level radiation exposure. It is a useful and convenient screening equipment to assess the bone health in schoolchildren [24].

Although the outcomes of the present study are promising, it has several limitations that need to be considered. One such limitation, we were unable to determine the relationship between puberty and BUA among the schoolchildren. It was not assessed due to facilities constrains, as the present study was conducted in schools. Second, although QUS device is a useful and valid method to assess the bone health in schoolchildren [50], DXA remains the gold standard assessment of bone density. However, the present study did not apply DXA as this method is expensive and exposes schoolchildren to ionizing radiation. As there is a number of evidence proposed that QUS device provides structural information that is relevant to the quality rather than the density of a bone alone [50], it is sensible to understand the factors correlated with bone properties as measured by QUS, e.g., lifestyle factors and dietary intake, to further justify the clinical value of this QUS technique [22].

## 5. Conclusions

On the whole, the findings from the present study demonstrated that Malaysian schoolchildren had significantly higher BUA as compared to Colombian, Nigerian and Spanish schoolchildren. Our findings revealed that body composition variables—including weight, height, body fat percentage, fat mass, fat free mass, visceral fat and waist circumference—were positively correlated with bone outcome variables, including BUA and Z-score. Understanding the relationship between adiposity and bone health may lead us to develop new therapeutic interventions to prevent both childhood obesity and osteoporosis. Since a number of studies have shown a positive effect of adiposity on bone health, further clinical and molecular studies in the future is recommended to fully illustrate the complex and captivating interactions between adiposity and bone health.

## Figures and Tables

**Table 1:** Demographic characteristics, anthropometric and bone outcome variables of schoolchildren (*n* = 415).

	Total (*n* = 415)		Boys (*n* = 196)		Girls (*n* = 219)	
	Mean ± SD	Median ± IqR	Mean ± SD	Median ± IqR	Mean ± SD	Median ± IqR
**Age** (year) ^⸸^	10.9 ± 0.8	10.8 ± 1.3	10.9 ± 0.8	10.8 ± 1.2	10.9 ± 0.8	10.8 ± 1.3
**Ethnicity**	*n* (%)		*n* (%)		*n* (%)	
Malay	277 (66.7)		129 (65.8)		148 (67.6)	
Chinese	103 (24.8)		55 (28.1)		48 (21.9)	
Indian	34 (8.2)		11 (5.6)		23 (10.5)	
Others	1 (0.3)		1 (0.5)		0	
**Anthropometric**						
Weight (kg);	37.9 ± 0.8	34.9 ± 14.8	37.7 ± 13.9	33.3 ± 15.1	38.1 ± 11.9	36.0 ± 14.0
Height (cm) ^⸸⸸^	142.9 ± 8.9	142.8 ± 12.0	141.8 ± 12.8	141.8 ± 12.7	143.5 ± 12.4 *	143.5 ± 12.4
Body fat (%) ^⸸^	23.9 ± 10.5	21.2 ± 14.8	22.5 ± 11.3	18.9 ± 17.6	25.2 ± 9.5 ***	23.7 ± 13.
Fat mass (kg) ^⸸^	10.1 ± 8.0	7.2 ± 8.3	9.7 ± 8.7	6.5 ± 8.8	10.5 ± 7.3 ***	8.3 ± 7.7
Fat free mass (kg) ^⸸^	27.8 ± 6.0	26.9 ± 7.7	28.0 ± 6.6	26.7 ± 7.9	27.6 ± 5.5	27.0 ± 7.4
Visceral fat ^⸸^	4 ± 4	3 ± 3	4 ± 4	2 ± 4	5 ± 4 ***	3 ± 3
WC (cm) ^⸸^	64.1 ± 12.5	61.0 ± 15.1	63.8 ± 13.3	69.8 ± 15.4	64.4 ± 11.8	61.4 ± 15.5
BMI z-score ^⸸^	−0.2 ± 2.3	−0.2 ± 2.3	−0.2 ± 3.1	−0.2 ± 3.1	−0.1 ± 1.9	−0.1 ± 1.9
**Bone health**						
BUA (dB/MHz) ^⸸⸸^	69.5 ± 7.3	69.5 ± 9.5	69.0 ± 7.4	69.2 ± 9.8	70.0 ± 7.2	69.9 ± 9.5
Z-score ^⸸⸸^	1.4 ± 1.4	1.4 ± 1.7	1.1 ± 1.0	1.0 ± 1.7	1.7 ± 1.3 ***	1.7 ± 1.7

IqR: interquartile range; SD: standard deviation; WC: waist circumference; BMI z-score: body mass index-for-age z-score; BUA: broadband ultrasound attenuation; ^⸸^ Mann-Whitney test; ^⸸⸸^ Independent *t*-test; * Significant at the 0.05 level; *** Significant at the 0.001 level.

**Table 2:** Comparison of mean broadband ultrasound attenuation (BUA) for schoolchildren from Malaysia (present study), Singapore, Taiwan, Colombia and Spain by sex and age.

Age (Years)	BUA of Boys (dB/MHz); Mean ± SD					BUA of Girls (dB/MHz); Mean ± SD				
	Malaysia *n* = 196	Singapore *n* = 417	Colombia *n* = 445	Nigeria *n* = 494	Spain *n* = 121	Malaysia *n* = 219	Singapore *n* = 333	Colombia *n* = 556	Nigeria *n* = 522	Spain *n* = 124
9.0–9.9	68.7 ± 6.3	57.2 ± 10.5	48.6 ± 9.2	56.4 ± 14.8	57.9 ± 12.4	68.7 ± 6.3	60.4 ± 11.0	51.8 ± 8.9	53.5 ± 12.7	53.1 ± 9.5
10.0–10.9	67.9 ± 8.2	63.4 ± 12.7	52.9 ± 10.4	58.9 ± 12.7	62.2 ± 13.0	68.9 ± 7.1	62.2 ± 9.5	51.7 ± 10.6	53.0 ± 11.1	60.8 ± 14.7
11.0–11.9	70.5 ± 7.0	69.7 ± 14.4	57.2 ± 10.1	63.5 ± 15.9	60.6 ± 14.0	71.4 ± 6.5	68.9 ± 13.6	60.5 ± 13.3	60.0 ± 17.6	59.6 ± 16.3
12.0–12.9	70.3 ± 4.5	75.4 ± 13.0	60.6 ± 10.5	63.5 ± 16.6	63.3 ± 14.7	71.4 ± 9.9	75.4 ± 19.4	63.7 ± 14.4	57.6 ± 13.3	64.7 ± 13.5

BUA: broadband ultrasound attenuation; SD: standard deviation; Malaysia vs. Nigeria, *p* < 0.001; Malaysia vs. Colombia, *p* < 0.001; Malaysia vs. Spain, *p* = 0.002; Singapore vs. Nigeria, *p* = 0.008; Singapore vs. Colombia, *p* < 0.001; One-way Anova and Tukey tests were applied.

**Table 3:** Unadjusted correlations of body composition variables with bone outcome variables in schoolchildren (*n* = 415).

	BUA		Z-Score	
	*r*-Value	*p*-Value	*r*-Value	*p*-Value
Weight (kg)	0.212	<0.001 ***	0.179	<0.001 ***
Height (cm)	0.317	<0.001 ***	0.289	<0.001 ***
Body fat percentage (%)	0.135	0.006 **	0.131	0.007 **
Fat mass (kg)	0.149	0.002 **	0.126	0.010 **
Fat free mass (kg)	0.251	<0.001 ***	0.212	<0.001 ***
Visceral fat	0.139	0.005 **	0.123	0.012 *
Waist circumference (cm)	0.167	0.001 **	0.149	0.002 **
BMI z-score	0.163	0.001 **	0.148	0.003 **

BUA: broadband ultrasound attenuation; BMI z-score: body mass index-for-age z-score; * Significant at the 0.05 level; ** Significant at the 0.01 level; *** Significant at the 0.001 level; Pearson correlation coefficient test was applied.

**Table 4:** Linear regression analysis models for bone outcome variables predicted by body composition variables.

	BUA			Z-Score		
	β Coefficient	95% CI	*p*-Value	β Coefficient	95% CI	*p*-Value
Weight (kg)	0.172	0.042, 0.153	0.001 **	0.160	0.007, 0.027	0.001 **
Height (cm)	0.299	0.153, 0.337	<0.001 ***	0.310	0.031, 0.065	<0.001 ***
Body fat percentage (%)	0.131	0.025, 0.158	0.007 **	0.104	0.001, 0.026	0.032 *
Fat mass (kg)	0.130	0.032, 0.205	0.007 **	0.107	0.002, 0.034	0.026 *
Fat free mass (kg)	0.209	0.127, 0.378	<0.001 ***	0.218	0.026, 0.073	<0.001 ***
Visceral fat	0.127	0.059, 0.401	0.008 *	0.107	0.004, 0.068	0.026 *
Waist circumference (cm)	0.165	0.042, 0.151	0.001 **	0.145	0.006, 0.026	0.002 **
BMI z-score	0.162	0.297, 1.125	0.001 **	0.150	0.047, 0.200	0.002 **

BUA: broadband ultrasound attenuation; BMI z-score: body mass index-for-age z-score; CI: confidence interval; All the models adjusted for age, sex and ethnicity; Sex: “0” represent boys and “1” represents girls; Ethnicity: “0” represents Malay and “1” represents Chinese, Indian and other ethnicity. Dependent variables: BUA and Z-score. Independent variables: weight, height, body fat percentage, fat mass, fat free mass, visceral fat, waist circumference and BMI z-score. Model assumptions were fulfilled; no multicollinearity detected; * Regression is significant at the 0.05 level; ** Regression is significant at the 0.01 level; *** Regression is significant at the 0.001 level.

## Data Availability

The data presented in this study are available on request from the corresponding author. The data are not publicly available due to ethical issue.

## Appendix A

**Table A1:** Comparison of mean height for schoolchildren from Malaysia (present study) and Colombia by sex and age.

Year	Height of Boys (m); Mean ± SD		Height of Girls (m); Mean ± SD	
	Malaysia; *n* = 196	Colombia; *n* = 445	Malaysia; *n* = 219	Colombia; *n* = 556
9.0–9.9	1.35 ± 0.06	1.32 ± 0.05	1.36 ± 0.07	1.34 ± 0.07
10.0–10.9	1.39 ± 0.08	1.37 ± 0.07	1.42 ± 0.09	1.39 ± 0.07
11.0–11.9	1.44 ± 0.07	1.43 ± 0.08	1.47 ± 0.06	1.42 ± 0.01
12.0–12.9	1.52 ± 0.08	1.47 ± 0.10	1.51 ± 0.07	1.47 ± 0.07
